# Association of Sexual Health and Mental Health in Erectile Dysfunction: Expert Opinion From the Indian Context

**DOI:** 10.7759/cureus.77851

**Published:** 2025-01-22

**Authors:** Satya S Vasan, Sanjay Pandey, Sathyanarayana T S Rao, Deepak M Gupte, Rahul R Gangavaram, Ajit Saxena, Rajiv Kovil, Praveen Joshi, Rajiv Goel, Sanjay K Mittal, Rajarshi Neogi, Sam P Joseph, Dhara Shah, Zenifer Khan

**Affiliations:** 1 Urology and Andrology, Ankur Hospital, Bangalore, IND; 2 Urology and Andrology, Kokilaben Hospital, Mumbai, IND; 3 Psychiatry, Jagadguru Sri Shivarathreeshwara Medical College, Jagadguru Sri Shivarathreeshwara Academy of Higher Education and Research, Mysore, IND; 4 Urology and Andrology, Shree Gurukrupa Clinic, Mumbai, IND; 5 Urology and Andrology, Androcare Andrology Institute, Hyderabad, IND; 6 Urology, Indraprastha Apollo Hospitals, Delhi, IND; 7 Diabetology, Kovil’s Diabetes Care Center, Mumbai, IND; 8 Urology and Andrology, Joshi’s Urology and Andrology Center, Bangalore, IND; 9 Urology, Manipal Hospital, Gurgaon, IND; 10 Urology, Santokba Durlabhji Memorial Hospital, Jaipur, IND; 11 Psychiatry, Radha Gobinda Kar Medical College, Kolkata, IND; 12 Psychiatry, Elite Mission Hospital, Thrissur, IND; 13 Medical Affairs and Pharmacology, Mylan Pharmaceuticals Private Limited, A Viatris Company, Bangalore, IND; 14 Pharmacology and Medical Affairs, Mylan Pharmaceuticals Private Limited, A Viatris Company, Bangalore, IND

**Keywords:** anxiety, depression, diabetes mellitus-induced erectile dysfunction, endothelial dysfunction, erectile dysfunction, mental health, phosphodiesterase-5 inhibitors, psychosexual therapy

## Abstract

Erectile dysfunction (ED) is a common condition in men, driven by a complex interplay of organic, relational, and psychological factors, necessitating an integrated treatment approach. Psychological factors, such as anxiety, depression, and stress, are significant contributors to erectile problems. Erectile dysfunction can have severe psychological consequences, including feelings of emasculation, humiliation, reduced self-confidence, isolation, loneliness, and a decline in overall well-being. A national advisory board comprising 12 experts from India, including 9 urologists and 3 psychiatrists, convened to discuss a multidisciplinary approach to the treatment of ED. Using a modified Delphi method and literature review, the 34 panels developed evidence-based insights. Experts highlighted the importance of thorough assessments of sexual dysfunction in patients. Given the frequent comorbidity of mental health issues with ED, physicians should proactively explore patients’ sexual and mental health. Creating a secure and welcoming environment is crucial for these assessments. Physicians should gather detailed information on psychological symptoms, stressors, relationship dynamics, cognitive style, and distractions. Experts highlighted the importance of thorough diagnostic assessments and recommended a multidisciplinary approach integrating pharmacological interventions (e.g., phosphodiesterase-5 inhibitors) with psychometric therapy, tailored to the age, existing comorbidities, and underlying causes of ED. A balanced, interdisciplinary approach incorporating psychosexual therapy, lifestyle modifications, and advanced therapies is crucial for the holistic management of ED. Key consensus recommendations also emphasized fostering open communication between patients and healthcare providers, routine mental health screenings in patients with ED, and early referrals to specialists when necessary. Clinicians should actively involve mental health professionals in the management of ED and prioritize individualized treatment strategies tailored to each patient’s needs. This multifactorial condition requires coordinated efforts to address both organic and psychogenic causes, restore patients’ quality of life, and promote open communication. By proactively engaging with patients, addressing their concerns, and facilitating referrals as needed, clinicians can significantly improve outcomes for patients with ED.

## Introduction and background

**Figure 1:**
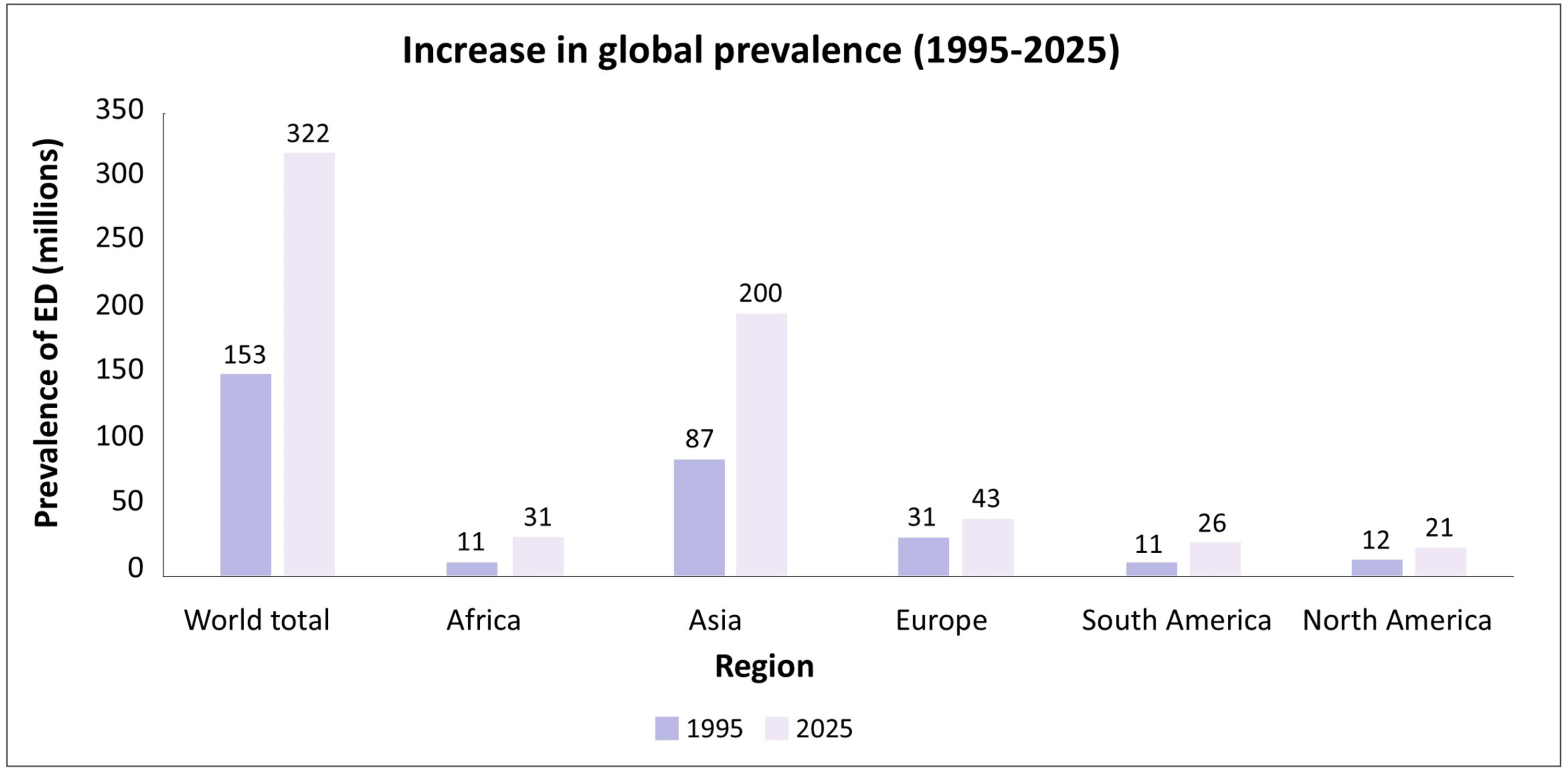
Probable global increase in the prevalence of erectile dysfunction from 1995 to 2005 ED: erectile dysfunction Based on data from: [2]

Penile erection is a complex process that requires coordination among the hormonal, vascular, and neural systems, with significant psychological input. Disruptions in any of these systems can lead to erectile dysfunction (ED) [1]. Erectile dysfunction is the consistent and recurrent inability or difficulty to attain or sustain an erection of sufficient rigidity and duration for satisfactory sexual intercourse. It is the most common complaint among men treated for sexual disorders, affecting over 50% of patients. Global estimates project that by 2025, ED will affect 322 million men worldwide, with a significant increase in the developing world (Figure 1) [2].

**Figure 2:**
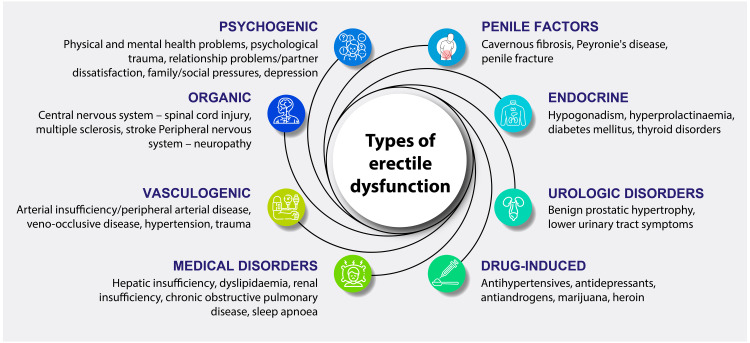
Factors contributing to erectile dysfunction Based on data from: [6]

Despite its prevalence, data on ED in India remain limited due to societal stigma, with many men reluctant to report the condition, complicating prevalence estimates [3,4]. A study from Southern India reported a prevalence of 15.8% [5]. Erectile dysfunction was found to be most common in the 51 to 60-year age group, with various factors contributing to the condition, including diabetes mellitus, hypertension, and smoking (Figure 2) [5,6].

Studies indicate that 61.4% of patients with diabetes mellitus [7,8], 30% of patients with hypertension, 42% of patients with hyperlipidemia, and 74% of patients with chronic obstructive pulmonary disease experience ED [9-13]. There is also a higher likelihood of severe ED in young, heavy smokers [14]. The prevalence of ED is increasing among younger men, as highlighted by a US study reporting mild ED in 11.3% and moderate-to-severe ED in 2.9% of sexually active men aged 18 to 31 years [15]. Psychosocial factors, such as depression and anxiety, further exacerbate the condition, affecting patients’ quality of life [16-18].

Other major risk factors that contribute to ED are age, a sedentary lifestyle with a lack of exercise, and obesity. Certain medications, such as antidepressants (e.g., selective serotonin reuptake inhibitors), cimetidine, ketoconazole, spironolactone, sympathetic blockers (e.g., methyldopa, clonidine, guanethidine), thiazide diuretics, and certain antihypertensives, can also contribute to the development of ED [19].

Another critical factor contributing to ED is endothelial dysfunction. This condition, marked by vascular damage, hinders endothelium function and the release of nitric oxide (NO), thereby affecting the relaxation and expansion of the blood vessels [18]. Endothelial dysfunction significantly contributes to ED by reducing the availability of NO, a critical mediator of smooth muscle relaxation in the penile vasculature [20,21]. Damage to the endothelium caused by conditions such as hypertension, diabetes, hyperlipidemia, or smoking results in oxidative stress, and compromised blood flow to penile tissue. Over time, the persistence of these factors exacerbates vascular damage, making endothelial dysfunction a pivotal mechanism underlying ED [20,21]. Preventing or mitigating endothelial dysfunction can significantly reduce the risk of ED. Lifestyle modifications, such as adopting a heart-healthy diet, engaging in regular aerobic exercise, quitting smoking, and reducing alcohol consumption can improve endothelial health [22,23]. Emerging therapies targeting endothelial repair hold promise for directly addressing ED. Vascular endothelial growth factor (VEGF), a critical mediator of vascular regeneration and angiogenesis, has gained significant attention as a potential treatment for ED caused by endothelial dysfunction [24]. VEGF stimulates the formation of new blood vessels and repairs damaged endothelium, thereby enhancing penile blood flow and erectile function. Research has shown that VEGF can alter physiological pathways associated with endothelial cell proliferation, which is closely linked to ED [25]. Preclinical studies have shown VEGF’s ability to restore vascular health in animal models of ED, particularly those with diabetes-induced endothelial damage. Although clinical studies evaluating VEGF are still in their early stages, they show encouraging results. Furthermore, low-intensity extracorporeal shock wave therapy has shown the potential to stimulate VEGF expression, leading to improved penile blood circulation and vascular health [26]. However, several challenges remain, including ensuring targeted delivery of VEGF, minimizing systemic side effects, and addressing its short biological half-life. Future research is directed at developing advanced delivery systems, such as nanoparticles or gene therapy, to enhance the safety, efficacy, and clinical applicability of VEGF in the management of ED [27,28].

This paper presents expert opinion from an interdisciplinary panel of specialists in India, along with a review of literature, providing insights into the current understanding of ED, its presentation, and recent trends in treatment. It emphasizes a multidisciplinary approach to address both sexual and psychological well-being in the Indian context.

## Review

A panel of 12 interdisciplinary specialists from India, including 9 urologists and 3 psychiatrists, convened an expert group in a national advisory board meeting held in November 2023. The purpose of the meeting was to understand the current landscape of ED, the diagnostic and treatment modalities, and the psychological impacts that warrant a multidisciplinary treatment approach. The insights were gathered using a three-step modified Delphi method [29]. The first step was an email-based questionnaire designed to assess key areas of management of ED by analyzing expert responses to identify preliminary points of agreement and divergence. This was followed by a face-to-face meeting, where experts presented arguments, discussed points from the previous round, and refined consensus statements. Key topics of disagreement such as the prioritization of advanced therapies like platelet-rich plasma and stem cell treatments were revisited, with conditional recommendations made as necessary. Consensus was defined as at least 75% agreement among experts on specific recommendations while areas lacking consensus were documented and excluded from the final recommendations. A final round of discussion was conducted over email to confirm consensus on unresolved points and finalize the recommendations. After a thorough discussion of various aspects of the association between mental health and ED, a conclusion was drawn. Additionally, a comprehensive literature search was conducted using scientific databases, such as PubMed and Google Scholar, with search terms related to ED and its association with mental health such as “erectile dysfunction”, “erectile disorder”, “erectile difficulties”, “erectile problems paired with mental health”, “psychogenic paired with mental health”, “anxiety in ED”, and “depression in ED”. The search included studies published in English and those reporting human clinical data, ensuring a focused and comprehensive review. Time limitations were not applied, allowing the inclusion of all pertinent studies in this area of critical need.

Patient-related barriers in erectile dysfunction management

Despite the high prevalence of ED and its negative impact on quality of life, it remains significantly underreported due to societal taboos, stigma, and fear surrounding sexual dysfunction [4,30,31]. Many men avoid seeking treatment because of embarrassment, difficulties communicating with their partners, low self-confidence, and fear of negative reactions [17,32]. This underreporting is further compounded by a misconception that ED is a normal part of aging, which discourages medical intervention [30,33]. Further, physicians often fail to proactively inquire about ED, resulting in underdiagnosis and limited understanding of its comorbidities [34]. Open dialog and proactive questioning by physicians are essential for improving the diagnosis and management of ED [34,35]. Physicians must also keep certain guiding principles in mind to facilitate these discussions (Table 1) [30].

**Table 1:** Guiding principles for physicians to treat patients with erectile dysfunction ED: erectile dysfunction; PDE-5Is: phosphodiesterase-5 inhibitors Based on the author’s personal experiences

Guiding principles	Explanation/example
Establish good patient rapport.	Creating a trusting relationship encourages patients to discuss sensitive issues like ED openly. For instance, beginning the conversation with open-ended questions, such as “Have you experienced any difficulties in your sexual life recently?” can help ease patients into the discussion.
Understand the patient’s ongoing psychological issues.	Identify and address mental health concerns contributing to ED. For example, a patient reporting symptoms of anxiety or depression may benefit from referral to a mental health professional while initiating PDE-5Is.
Be flexible and cognizant of the deep-seated opinions and values of each patient, including cultural and religious beliefs.	Tailor discussions to respect individual beliefs. For instance, in conservative communities, avoid medical jargon, and explain ED in a culturally sensitive manner, emphasizing its treatable nature.
Prefer a quiet environment without any interruptions or a sense of haste during initial discussions.	Allocate dedicated consultation time in a private setting, ensuring the patient feels comfortable discussing intimate concerns without embarrassment or judgment.
Discuss the sexual terminology in layman’s language.	Replace medical terms with simpler language. For example, instead of “erectile dysfunction” use “difficulty in maintaining an erection.”
Consider interviewing the partner with the patient's consent.	Understanding the partner's perspective can provide insights into relationship dynamics. For example, if a patient consents, involve the partner in discussions about treatment options and lifestyle changes.

A 2019 study reported that nearly 70% of men with ED did not seek treatment, citing cultural taboos and fear of judgment as primary barriers [36]. Another global survey reported that only 16.7% of men with ED discuss their condition with a healthcare provider, often leading to delayed diagnosis and management [37]. These findings emphasize the need for public health campaigns and physician engagement to normalize conversations around ED.

• *Expert Opinion: Societal Taboo Around Sexual Dysfunction*

The underreporting of ED is a result of societal stigma, fear, and misconceptions surrounding the condition. To address this issue, it is crucial to promote open dialogue, share accurate information, and encourage healthcare providers to actively inquire about ED during patient consultations. By doing so, we can improve the diagnosis, treatment, and overall management of this common yet complex problem.

Role of diabetologists in treating patients with erectile dysfunction

The prevalence of ED in men with type 2 diabetes mellitus (T2DM) ranges from 20% to 85% [38]. India, currently ranking second in the global diabetes epidemic after China, has seen a notable increase in diabetes mellitus-induced erectile dysfunction (DMED) [39,40]. A study conducted in Northern India found that 67.4% of individuals with T2DM experienced ED, with 42.4% of these cases classified as severe [4]. Similarly, another study from Jammu, India, reported a 62.1% prevalence of ED among patients with T2DM [41]. These statistics underscore the need for Indian diabetologists to integrate the assessment and management of ED into routine T2DM care. Early diagnosis and treatment are essential to manage risk factors and improve the quality of life for these patients.

• *Expert Opinion: Involving Mental Health Professionals in ED Management*

Diabetologists in India should be trained to recognize and address DMED effectively, ensuring timely diagnosis and comprehensive care. If a physician believes that the patient is experiencing sexual dysfunction, it is advisable to seek the expertise of a mental health professional. Mental health professionals should promote treatment adherence, reduce performance anxiety, and integrate treatments into a sexual relationship.

Molecular basis of erectile dysfunction

The peripheral nervous system, including sympathetic, parasympathetic, somatic, and sensory innervation, regulates penile erection. These nerves, along with vascular endothelium, release transmitters and modulators that control the contractile state of penile smooth muscles [42]. The corpus cavernosum, lined with vascular endothelium, plays a crucial role in penile tumescence by facilitating blood flow through penile arteries. Abnormalities in the penile vasculature or disruptions in the neurovascular processes can lead to ED [43]. Studies have shown that the administration of VEGF, a mitogen specific to endothelial cells, can improve dysfunction in both endothelial and smooth muscle cells in animal models of ED [44,45]. VEGF supports endothelial repair and enhances blood flow, which is essential for proper erectile function. By improving vascular endothelial function, VEGF-based therapies offer the potential for restoring smooth muscle relaxation and improving penile rigidity during erection.

• *Expert Opinion: ED Management Based on Its Cause*

There are two distinct causes of ED, namely, organic and psychogenic. It is crucial for healthcare professionals to comprehend the underlying cause of ED so that they can effectively refer individuals to appropriate specialists for precise diagnosis and treatment.

Multidisciplinary treatment approaches should be considered for the management of ED. If psychological treatment is considered necessary, patients should be referred to a psychiatrist. Conversely, psychiatrists should refer patients with ED to a urologist for evaluation.

Assessment and diagnostic approach for erectile dysfunction

**Figure 3:**
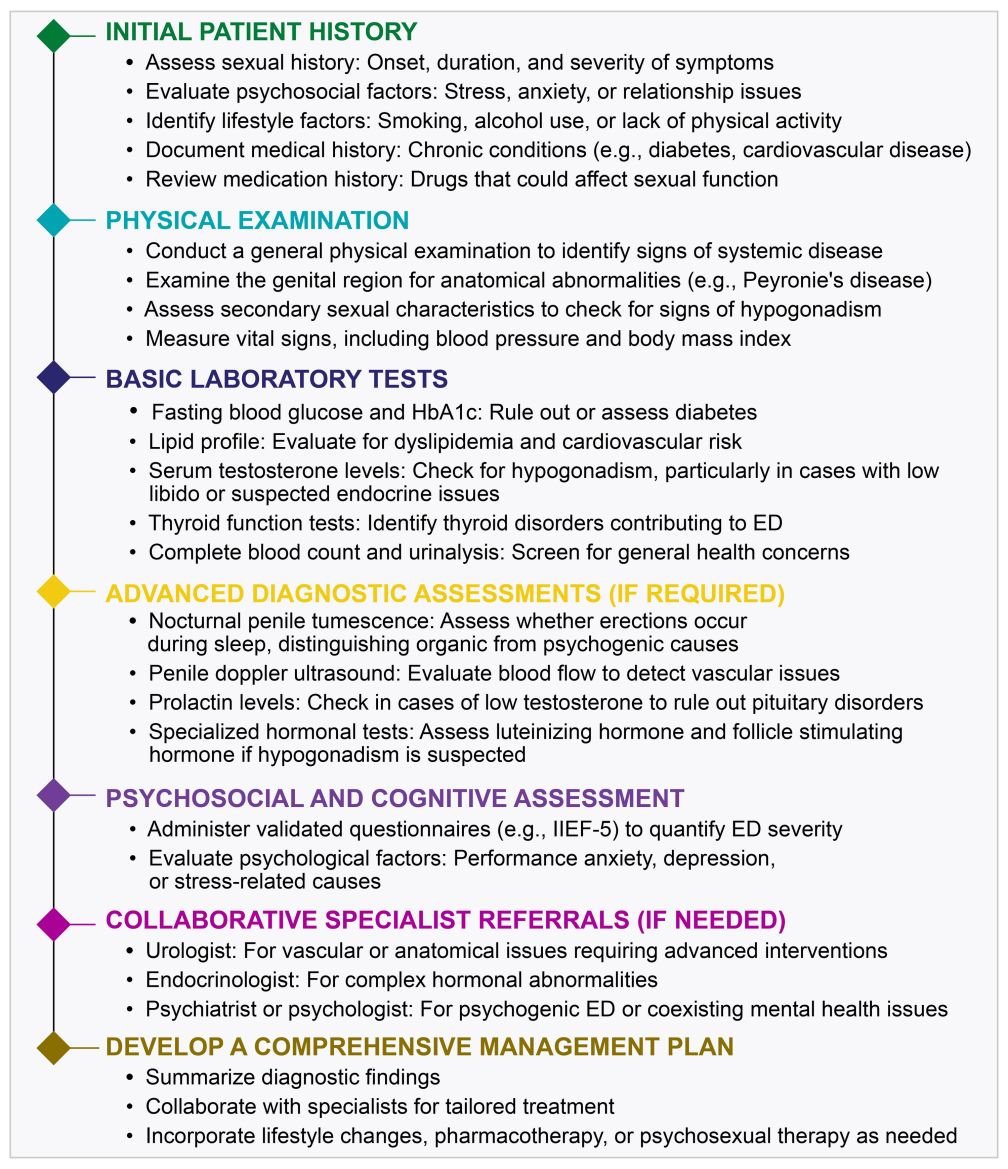
Comprehensive diagnostic pathway for erectile dysfunction ED: erectile dysfunction; HbA1c: glycated hemoglobin; IIEF: International Index of Erectile Function Based on the author’s personal experiences

A comprehensive evaluation of ED requires both physical and psychological assessments. General and specific physical examinations, along with validated questionnaires, such as the International Index of Erectile Function (IIEF), are recommended to assess the severity of ED. Laboratory tests should include hormone analysis, such as testosterone levels, to identify underlying causes. The recommended assessments for ED are listed below. To streamline the assessment process, a comprehensive diagnostic pathway for ED is summarized in Figure 3, outlining each step from history-taking to advanced diagnostic evaluations.

General Physical Assessment Including Psychological Assessment

A thorough medical history review should be conducted, including details regarding the onset and duration of ED, correlation with specific partners, conflicts between the couple, quality of erections or rigidity, nocturnal erections, and ejaculation patterns [46,47].

Cardiovascular risk factors (e.g., hypertension, diabetes), neurological symptoms (e.g., numbness, weakness), and medication history focusing on drugs that can affect erectile function (e.g., beta-blockers, antidepressants) should be evaluated. Specific inquiries about prior surgeries (e.g., pelvic or penile), lifestyle factors (e.g., smoking, alcohol use), and psychological history (e.g., anxiety, depression, relationship issues) should also be considered.

A general and specific physical examination should be performed. The genital examination should identify abnormalities, such as penile plaques or curvature (Peyronie’s disease), signs of testosterone deficiency (e.g., gynecomastia, underdeveloped secondary sexual characteristics), or testicular abnormalities (e.g., atrophy, masses).

Physical examination should include vascular assessment (e.g., peripheral pulses), neurological evaluation (e.g., perineal reflexes), and genital measurements.

An evaluation of the patient's lifestyle should be undertaken, which should include a review of sleep quality and duration, the presence of snoring or sleep apnea, body weight, exercise habits, alcohol intake, and smoking history. Moreover, it is crucial to assess the patient’s overall health, including both physical and mental well-being, as well as any current medications. A physical examination of the lower genitourinary tract, penis, and testicles should also be conducted, in addition to exploring the patient’s relationship and psychosexual history.

The psychosocial history should include aspects such as sexual education, sexual orientation, a history of abuse, sexual distress, and the presence of comorbid conditions like anxiety and depression. It is also essential to evaluate factors, such as sexual desire, subjective sexual arousal, adequate stimulation, cognitive distraction, automatic thoughts, perfectionism, negative effects, and neuroticism [19,48].

A detailed history of substance abuse should be obtained. Excessive alcohol and drug use can cause sexual dysfunction by affecting the penile neurovascular system, increasing prolactin release, and reducing testosterone production [49].

Treatment and medication history should be thoroughly reviewed, including prescribed drugs, over-the-counter medications, and culturally approved aphrodisiacs [50].

Assessments for hypogonadism, loss of libido, depression with low mood, and other psychological conditions should be conducted to ensure a comprehensive understanding of contributing factors [17].

Specific Physical Assessment

Specialized tests are recommended when history and physical examination findings suggest further evaluations:

Nocturnal penile tumescence and rigidity: This test should be conducted for patients with suspected psychogenic ED [50]. Preserved nocturnal erections may negate the need for invasive vascular testing.

Rigiscan monitoring: This should be used to track nocturnal penile tumescence [50].

Dynamic infusion cavernosometry and cavernosography: These may be used selectively to evaluate venous ED [51].

Pharmaco penile duplex ultrasonography: This test should be conducted for patients with suspected vascular causes of ED (e.g., arterial insufficiency, venous leak) or prior penile trauma [52]. It provides a detailed evaluation of penile blood flow and vascular structures.

Hormonal assessments: These are recommended for patients presenting with symptoms of hypogonadism (e.g., low libido, reduced energy) or systemic signs of endocrine disorders [53,54]. Morning testosterone levels, along with luteinizing hormone, and prolactin, should be measured.

Other physical assessments: These include color Doppler ultrasound, selective internal pudendal arteriography by digital subtraction angiography, dual-energy CT arteriography (D-e CTA), penile cavernosography, and ​​​320-detector row dynamic volume CT (4D-CTA) [55-59].

Assessment Using Questionnaires

Questionnaires such as the IIEF-15 or IIEF-5 should be employed for further assessment. A score below 26 on the IIEF-15 or below 22 on the IIEF-5 indicates ED. The severity of ED can be categorized as mild, moderate, or severe based on these scores [60].

Laboratory Tests

Follicle-stimulating hormone, dehydroepiandrosterone sulfate​​​​​, estradiol, prolactin, and luteinizing hormone should be analyzed [61].

A morning testosterone level measurement should be conducted to identify hormonal imbalances [62].

• *Expert Opinion: Tools for Assessing ED and Mental Health*

Newer tools, such as vascular endothelial growth factor testing, can be utilized. A simple blood examination can help early detection of sexual dysfunction.

Validated questionnaires are recommended to assess the severity of ED, to measure treatment effectiveness, and to guide future management. A short-form validated questionnaire may be most appropriate; examples include the Erection Hardness Score.

Patients can be given a tool to address sexual dysfunction inquiries by scanning a QR code at the physician's office. The responses would be securely sent to the doctor for review before the consultation, allowing them to offer appropriate treatment recommendations.

Erectile dysfunction and psychological well-being

Impact of Mental Health on ED

Erectile dysfunction can profoundly affect psychological and emotional well-being, not only for individuals but also for their partners. A thorough evaluation and effective management of ED are essential [17,63]. A meta-analysis showed that individuals with depression are at a higher risk of ED and vice versa [64]. Additionally, an analysis of 12 studies found a three-fold increase in the likelihood of developing depression in individuals with ED [65].

Beyond depression, ED is linked to anxiety disorders, including post-traumatic stress disorder, obsessive-compulsive disorder, social phobia/social anxiety disorder, and panic disorder. Erectile dysfunction often triggers feelings of humiliation, shame, and fear of abandonment, further exacerbating feelings of inadequacy and guilt [31]. Specific psychological conditions, such as depression, anxiety, and performance anxiety, significantly influence ED [66,67]. Depression impacts libido and sexual arousal [68] while performance anxiety creates a cycle of stress and impaired sexual function, often leading to avoidance behaviors [69]. Interpersonal factors like unresolved conflicts or poor communication further impair erectile function, necessitating thorough psychological assessment to identify the contributing factors.

Approaches to Psychosocial Treatment

Incorporating psychological assessments into the routine evaluation of ED is essential for identifying underlying mental health issues such as anxiety and depression. Tools like the Mini-International Neuropsychiatric Interview and the Hospital Anxiety and Depression Scale offer quick insights into the patient’s psychological states, guiding further diagnostic and therapeutic interventions [70,71]. Psychological therapies, including cognitive behavioral therapy (CBT), play a crucial role in the management of ED.

Psychosocial treatments focus on four domains: (1) alleviating anxiety and desensitization, (2) implementing CBTs, (3) enhancing sexual stimulation, and (4) fostering interpersonal assertiveness and communication between couples [72]. CBT is particularly effective for addressing performance anxiety and negative thought patterns. Techniques like cognitive restructuring, gradual exposure, and relaxation exercises help patients reduce anxiety and build sexual confidence [73,74].

Combining CBT with pharmacological treatments, such as PDE-5Is, provides a comprehensive approach to managing ED. Studies report that combining CBT with sildenafil yields a 58% success rate and 45% satisfaction after four weeks [75]. Furthermore, a meta-analysis of randomized controlled studies reports average remission rates for psychogenic ED between 56% and 69% [76].

Psychosexual counseling has been shown to enhance relationship dynamics, communication, and emotional intimacy between partners [77]. Approaches like the sensate focus technique, which progresses from non-sexual touch to intimate contact, reduce performance anxiety and foster trust [78,79]. Couple counseling and psychosexual therapy have shown significant improvements in relationship satisfaction, addressing both sexual and relational conflicts [79,80]. In another study, psychosexual therapy showed superior improvements in sexual function compared to bupropion extended-release (BUP ER), highlighting its efficacy as an alternative to pharmacotherapy [81]. Research also indicates that sexual satisfaction contributes to 15% to 20% of overall relationship satisfaction while dissatisfaction in sexual function accounts for 50% to 70% of relationship dissatisfaction [82].

Effective communication between partners is crucial. Techniques such as active listening, scheduling non-sexual physical attention, and role-playing constructive dialogues during therapy sessions can improve emotional intimacy and relationship dynamics. Engaging partners in the management of ED through couples therapy fosters mutual understanding, reduces stigma, and enhances treatment outcomes [79]. By addressing both the psychological and relational aspects of ED, clinicians can provide a holistic approach to improving patient outcomes and relationship satisfaction.

• *Expert Opinion: ED and Psychological Well-Being*

Tools like the Mini-International Neuropsychiatric Interview and the Hospital Anxiety and Depression Scale are essential for identifying psychiatric disorders in patients with ED. Routine evaluations for anxiety, depression, and stress are crucial.

Depression and ED share a bidirectional link, with each increasing the risk of the other. Routine screening is vital for individuals at risk. Generalized and performance anxiety disrupts sexual function, creates a stress avoidance cycle, and strains communication. A thorough evaluation is essential to address these issues effectively.

Sexual dysfunctions related to desire, arousal, orgasm, and resolution may be caused by psychosocial factors. Psychosexual counseling, sex therapy, and CBT can address these within a biopsychosocial framework. Primary care physicians should assess psychological contributors like anxiety and refer patients to mental health professionals when needed.

ED can strain relationships, leading to dissatisfaction. Partner-focused therapies, such as couple counseling and the sensate focus technique, improve intimacy, communication, and treatment outcomes.

Combining psychological interventions like CBT with pharmacological treatments, such as PDE-5Is, leads to higher success rates and sustained improvements in ED, with remission rates reaching up to 69%. Addressing both psychological and relational factors through a multidisciplinary approach enhances outcomes for patients and their partners.

Strategies for the management of erectile dysfunction

**Figure 4:**
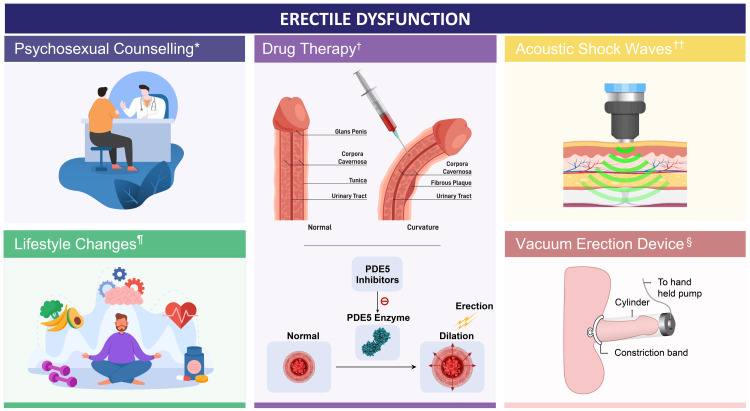
Multidisciplinary approach for the management of erectile dysfunction *Psychosexual counseling: address emotional and psychological factors; †Drug therapy: utilize medications to enhance erectile function; ††Acoustic shock wave: enhance vascularization and improve blood flow in the penis; ¶Lifestyle changes: promote healthy habits and physical activity; §Vacuum erection device: use vacuum devices to mechanically induce erections PDE5: phosphodiesterase type 5 Based on data from: [48]

Given the multifactorial nature of ED, a multidisciplinary approach is recommended (Figure 4) [81].

Clinicians can enhance this approach by conducting joint consultations, where specialists such as urologists, psychiatrists, and endocrinologists collaborate on complex cases to streamline diagnosis and treatment planning. For instance, a patient with ED and diabetes might benefit from simultaneous input from a diabetologist and a urologist. Shared electronic health records can play a crucial role in ensuring seamless communication among specialists by providing access to recent evaluations and test results. Establishing clear referral protocols, such as urologists referring patients with severe performance anxiety to psychologists, ensures comprehensive care and avoids treatment gaps. Regular interdisciplinary case discussions can provide a valuable platform for specialists to share insights and jointly address challenging cases. A comprehensive approach that integrates pharmacological interventions for organic causes with psychological therapies, such as CBT or psychosexual counseling for psychogenic factors, ensures that both biological and psychological dimensions of ED are effectively managed. This tailored strategy is particularly critical for optimizing patient outcomes across diverse age groups and health conditions. These strategies aim to improve patient outcomes but can also build confidence by demonstrating cohesive and collaborative care.

Lifestyle Modifications

Lifestyle changes play a critical role in managing ED. Regular aerobic physical activities, such as running, jogging, cycling, or swimming for 20-60 minutes per session with 3-5 sessions per week have been shown to improve vascular health and erectile function [82]. Another study found that supervised aerobic exercise of moderate to vigorous intensity, performed 4 times per week for 40 minutes over 6 months, may help reduce ED in men with arterial ED due to physical inactivity, obesity, hypertension, metabolic syndrome, and cardiovascular diseases [83]. Resistance training (e.g., weightlifting) performed for 20-40 minutes per session, 3 times per week, may also help enhance testosterone levels and overall fitness [84]. Patients should aim for a combination of these exercises tailored to their fitness levels and medical conditions.

Nutritional recommendations could focus on adopting a healthy diet, which includes whole grains, fresh fruits and vegetables, nuts, seeds, olive oil, lean proteins, such as fish and poultry, and limited consumption of red meat and processed foods. Patients with obesity might target a gradual weight loss of 10 to 20 kg over 6 months, as studies indicate this degree of weight loss can significantly improve ED [85,86]. Limiting alcohol consumption and avoiding sugary beverages are also recommended.

Smoking cessation programs, including behavioral therapy and pharmacologic aids like nicotine replacement or medications, such as varenicline, should be encouraged. Reducing sedentary behavior, managing stress through mindfulness or yoga, and improving sleep quality (targeting 7-9 hours per day) are additional lifestyle goals that may support the management of ED [87-89].

• *Expert Opinion: Tips for the Management of ED*

Lifestyle modification and pharmacotherapy can improve ED. Treating physicians should include experts from multidisciplinary fields, such as andrologists, sexologists, urologists, and psychiatrists, to address the biological and psychological aspects of ED. Physicians must thoroughly evaluate sexual disorders and identify underlying causes of ED. Medication should be pursued for treatable conditions, while psychological factors warrant referrals to psychiatrists. It is recommended to treat modifiable risk factors by managing diabetes, lowering hypertension, combating obesity, reducing adiposity, tackling metabolic syndrome, and implementing lifestyle modifications (e.g., exercising, quitting smoking, and avoiding a sedentary lifestyle).

Pharmacological Interventions

A holistic approach to the management of ED may involve combining psychosexual therapy with pharmacotherapy. While PDE-5Is like sildenafil are highly effective in improving erectile function, their limitations in addressing psychogenic ED and the potential side effects for certain populations must be considered. Psychosexual therapy can address the psychological factors that may contribute to ED, and when combined with pharmacotherapy, it offers a more comprehensive approach [90]. This integrated approach allows patients to address both the physical and emotional aspects of their condition. Healthcare providers are encouraged to assess the patient’s psychological state and consider recommending psychosexual counseling, such as CBT or sensate focus exercises, alongside pharmacological treatments. The combination can be particularly beneficial for patients with psychogenic ED and offers a more well-rounded treatment plan. Regular follow-up visits are essential to monitor progress and adjust treatment plans as needed.

Injectable Vasodilators

Several other peripherally acting agents, such as Maxi-K channel activators, guanylate cyclase activators, and NO donors, are being explored as potential therapies for ED [91]. Injectable intracavernous vasodilators, such as alprostadil, may be used as second-line treatments, either alone or in combination with agents like papaverine and phentolamine [92]. Intracavernous injections are particularly beneficial for patients who are unresponsive to oral PDE-5Is, with alprostadil acting directly on penile smooth muscle within 5-20 minutes. Combination regimens can increase efficacy but may also elevate the risk of side effects, such as penile pain or fibrosis [93].

Advanced Therapies

PDE-5Is such as sildenafil, vardenafil, avanafil, and tadalafil are commonly regarded as the first-line treatments for ED and remain a preferred option for its management [48]. These medications inhibit the PDE-5 enzyme, which inactivates cyclic guanosine monophosphate, leading to enhanced penile blood flow and improved erections [94,95]. Sildenafil, the first oral medication shown to significantly improve ED, has revolutionized ED management by demonstrating efficacy across a range of etiologies [96]. Studies indicate that sildenafil significantly increases the success rate of sexual intercourse attempts as compared to placebo (p<0.001), with success rates as high as 69% in the sildenafil group versus 22% in the placebo group [97]. A meta-analysis of double-blind, placebo-controlled studies revealed that patients taking sildenafil reported improved erections (46.5% to 87%) compared to placebo (11.3% to 41.3%). Furthermore, successful intercourse attempts were higher with sildenafil (52.6% to 80.1%) compared to placebo (14.0% to 34.5%) [98]. The long-term efficacy of sildenafil has been supported by clinical studies. The survey conducted after 12 months or longer demonstrated that 96% and 99% of patients reported satisfaction and improved sexual performance [99].

The various PDE-5Is differ in their pharmacological properties, allowing for tailored treatment options based on individual patient needs [100,101]. Sildenafil, for instance, has a rapid onset of action, taking effect in approximately 30 minutes, and remains effective for 4-6 hours, making it ideal for on-demand use. Tadalafil, often referred to as the “weekend pill,” begins acting within 20 minutes and has a long duration of up to 24-36 hours, allowing greater flexibility and spontaneity. Avanafil, with its quick onset of 15-30 minutes and effects lasting beyond 6 hours, is a practical choice for patients who prefer spontaneity [100]. Vardenafil, with an onset time of 10-30 minutes and a duration of 5 and 7 hours, is frequently recommended for individuals with coexisting metabolic conditions [102]. Potential side effects associated with PDE-5Is include headache, flushing, dyspepsia, and transient visual disturbances, particularly with sildenafil. Contraindications include the concurrent use of nitrates, and caution is necessary for patients with severe cardiovascular disease [101].

In addition to established pharmacological treatments, emerging therapies show promise for addressing the underlying causes of ED. Stem cell therapy is one such advancement, with studies suggesting its potential to promote angiogenesis and repair damaged endothelial tissues, particularly in cases of vascular ED. However, this approach has some challenges such as high costs, lack of standardization, and limited long-term safety data [103].

Another novel option is platelet-rich plasma therapy, which involves injecting plasma enriched with growth factors into the penile tissue to enhance vascular regeneration. Early studies have reported improvements in erectile function, but evidence remains limited and the therapy has yet to be standardized. Potential risk includes pain at the injection site and the need for repeated treatments [103].

Device-Based Approaches and Surgery

Advancements in penile prosthetics have also contributed to the management of ED. Modern prosthetics offer improved mechanical reliability and enhanced concealability, achieving patient satisfaction rates exceeding 90% [104]. Despite these advancements, prosthetic devices remain invasive and irreversible, making them a last-resort option for refractory ED cases [105]. Future directions in the treatment of ED involve ongoing research to improve the efficacy and safety of emerging therapies. For example, second-generation stem cell treatments utilizing genetically modified cells are being explored to enhance angiogenic potential [106]. Efforts are also underway to standardize platelet-rich plasma protocols by optimizing dosages and treatment intervals for maximum therapeutic benefit [107,108]. Additionally, advancements in artificial intelligence and 3D printing hold promise for further customization and improved outcomes in penile prosthetic designs [109]. While these innovations are promising, large-scale, randomized studies are necessary to validate their long-term benefits and establish standardized guidelines for their application.

Vacuum erection devices present another cost-effective and low-invasive treatment option for ED [110]. Emerging therapies like shockwave therapy, which targets the penis to improve vascularization, are also being explored. While robust evidence supporting its effectiveness remains limited, this therapy has shown promise in older individuals with vasculogenic ED [111].

• *Expert Opinion: First-Line Treatment for ED - PDE-5 Inhibitors*

PDE-5Is such as sildenafil are recommended as first-line therapy for ED. It is recommended to adjust the dose of sildenafil gradually, either by increasing or decreasing to determine an appropriate maintenance level. The initial dose should be titrated cautiously until the desired effect is achieved. Additionally, it is advisable to avoid taking the medication at night. Patients with comorbid conditions should be prioritized for the treatment of endothelial dysfunction. Co-administration of carnitine, arginine, and antioxidants with sildenafil can enhance its efficacy in managing ED.

Management variations for different populations

The management of ED must be tailored to specific patient demographics. For younger men with psychogenic ED, psychological interventions such as CBT or psychosexual counseling combined with PDE-5Is are often recommended. This approach addresses the psychological factors contributing to ED while improving physical outcomes. Older patients with multiple comorbidities require a more cautious approach. Lower doses of PDE-5Is and non-invasive treatments like vacuum erection devices may be preferred to minimize the risks associated with polypharmacy and age-related conditions such as cardiovascular disease. For patients with chronic illnesses, a multidisciplinary approach is essential, and older patients may benefit more from pharmacological and lifestyle interventions. Cultural considerations should also inform treatment plans, as understanding the impact of cultural attitudes and stigma around ED is essential to ensure effective and respectful care.

Long-term monitoring and follow-up

Effective management of ED extends beyond the initial treatment phase and requires ongoing monitoring to assess outcomes and adjust interventions as needed. Patients should be reevaluated 6-8 weeks after initiating therapy, with follow-up visits every 6-12 months to review treatment effectiveness, address any recurrence of symptoms, and assess patient satisfaction [112]. Follow-up assessments should also evaluate psychological well-being, adherence to lifestyle modifications, and potential side effects from pharmacological or device therapies. For patients on PDE-5Is, regular cardiovascular assessments are recommended to identify any underlying risks. Psychosexual outcomes, including improved relationship dynamics and reduced performance anxiety, should be measured using standardized tools such as the IIEF and patient satisfaction surveys.

• *Expert Opinion: Psychological Approach to Treating ED*

Physicians should assess barriers to ED treatment, such as low self-confidence, embarrassment, communication challenges between partners, and the stigma associated with ED and its management. Psychosexual therapy techniques, such as sensate focus, sex education, and interpersonal therapy, should be included to help reduce anxiety, enhance intimacy, and improve partner communication. Antidepressants should be carefully chosen and prescribed alongside ED treatments to address depression while minimizing the potential impact on sexual function. The inclusion of anxiolytics should be considered to manage anxiety and support better treatment outcomes in the patient-partner relationship.

Guidelines to manage erectile dysfunction

The American Urological Association (AUA) and European Association of Urology (EAU) guidelines offer comprehensive frameworks for managing ED. These guidelines emphasize the interplay between psychological and physical health (Table 2) [110,113].

**Table 2:** Guidelines for the management of erectile dysfunction. BUN: blood urea nitrogen; Cr: creatinine; ED: erectile dysfunction; HbA1c: glycated hemoglobin Source: [110,113]

2018 American Urological Association Guidelines	2023 European Association of Urology Guidelines
Risk factors	
Age, smoking, diabetes mellitus, hypertension, dyslipidemia, depression, obesity, and a sedentary lifestyle are the risk factors for ED	Age, diabetes mellitus, dyslipidemia, hypertension, cardiovascular disease, body mass index/obesity/waist circumference, metabolic syndrome, hyperhomocysteinemia, lack of exercise, and smoking are the risk factors. The consumption of thiazide diuretics, β-blockers, and psychotropic drugs are also related.
Depression, smoking, premature ejaculation, lower urinary tract symptoms secondary to benign prostatic hyperplasia, overactive bladder, age, and mental disorders are the other risk factors for ED. Other factors include vascular disease, tobacco use, neurologic disease, endocrinopathies, and medication-related side effects.	Atrial fibrillation, hyperthyroidism, vitamin D deficiency, hyperuricemia, folic acid deficiency, depression, anxiety disorders, chronic kidney disease, rheumatic disease, stroke, chronic obstructive pulmonary disease, migraine, and inflammatory bowel disease are related risk factors for ED.
Evaluation and diagnosis	
History-taking	
Men presenting with symptoms of ED should undergo a thorough medical, sexual, and psychosocial history.	A detailed sexual history including previous and current sexual relationships, emotional well-being, and current erectile problems with treatment and consultations taken before.
The medical history should also include comorbid medical and psychological conditions, prior surgeries, medications, family history of vascular disease, and substance use.	Patients should be screened for symptoms of possible hypogonadism, decreased energy and libido, and lower urinary tract symptoms. History of surgical intervention for cryptorchidism or hypospadias must be taken into account as possible signs of congenital defects.
Partner’s views on ED should be assessed, when possible. Additional details, such as the duration of the relationship, ongoing or unresolved interpersonal/relationship issues, the partner’s views on sexuality, and the partner’s personal health/sexual issues, should be considered for evaluation of ED.	Relationship factors, such as lack of satisfaction with the partner, poor sexual relationships, length of the relationship, or feeling emotionally disconnected from the partner during sex, should be considered a part of history. A history of use of drugs that interferes with the hypothalamus-pituitary gonadal axis should be noted.
Physical examination	
Men presenting with symptoms of ED should undergo a thorough medical, sexual, and psychosocial history; a physical examination; and selective laboratory testing.	Obesity is mostly associated with hypogonadism; hence, the determination of body mass index and the measurement of waist circumference are strongly recommended in all individuals.
Obesity is a key indicator of ED risk; thus, waist circumference should be checked. Physical examination should include assessment for signs of testosterone deficiency (e.g., gynecomastia, under-developed facial/pubic/axillary hair). Examination of the penis for occult deformities or plaque lesions should be done. The presence/absence of a palpable plaque is definitive evidence of penile deformity. The general consistency of the penile tissue should be checked along with a scrotal examination.	A physical examination of the genitourinary, endocrine, vascular, and neurological systems should be performed to specifically observe for Peyronie’s disease, pre or malignant genital lesions, prostatic enlargement/irregularity/nodularity, and signs of hypogonadism.
Specialized tests can be conducted on patients that may require detailed evaluation. Specialized tests include: 1. Nocturnal penile tumescence: Involves placement of two strain gauges on the penile shaft to measure radial rigidity during sleep Rigidity test and in-office testing: Assessing the veno-occlusive function of the penis wherein an erectogenic agent is injected into the corpora cavernosa of the penis. Duplex ultrasound is the gold standard in penile vascular evaluation.	Nocturnal penile tumescence and rigidity using Rigiscan vascular studies, intracavernous vasoactive drug injection, penile dynamic duplex ultrasonography, penile dynamic infusion cavernosometry and cavernosography, internal pudendal arteriography, specialized endocrinological studies, and specialized psycho-diagnostic evaluation.
Laboratory parameters	
Serum BUN/Cr, fasting lipids, fasting glucose or HbA1c, morning testosterone, thyroid function, and prostate-specific antigen.	Fasting blood glucose or HbA1c and lipid profile measurements should be conducted every year. A testosterone test (in a fasting state) including bioavailable or calculated-free testosterone should be performed. Prostate-specific antigen, prolactin, luteinizing hormone, and sex-hormone-binding globulin can be considered for screening of metabolic syndrome.
Questionnaires that can be dispensed to patients	
Erection hardness score	Sexual dysfunctional beliefs questionnaire
Sexual health inventory for men	Sexual modes questionnaire
International index of erectile function	Nil
Male sexual health questionnaire	Nil
Treatment	
For men being treated for ED, referral to a mental health professional should be considered to promote treatment adherence, reduce performance anxiety, and integrate treatments into a sexual relationship.	Psychological treatments should be considered and referring patients to a sexual therapist, psychologist, or psychiatrist is appropriate and often warranted.

Evaluation and diagnosis:The EAU highlights the importance of screening for metabolic syndrome and advanced diagnostic tools like vascular and endocrinological assessments while the AUA focuses on broader patient-reported outcomes

Risk factors: The EAU addresses regional conditions, such as hyperhomocysteinemia, and vitamin D deficiency, whereas the AUA emphasizes common risk factors like diabetes and smoking

Treatment recommendations**:** The EAU integrates emerging therapies, such as stem cell treatments, and highlights lifestyle interventions. In contrast, the AUA provides detailed guidelines on surgical solutions, like penile prostheses.

These differences reflect variations in healthcare priorities and patient demographics. The EAU emphasizes preventive care and advanced therapies, consistent with Europe’s broader access to metabolic and vascular screenings. In contrast, the AUA addresses comorbidities prevalent in the US, such as obesity and cardiovascular disease, with a focus on established treatment modalities. Cultural factors may also influence these guidelines. For example, the EAU addresses a higher prevalence of metabolic syndrome in Europe and a preventive approach to healthcare while the AUA reflects the US context, where there is greater emphasis on managing comorbidities and surgical interventions.

Future directions in the treatment of ED

Despite significant advancements in the management of ED, several gaps persist. Emerging therapies, such as VEGF and platelet-rich plasma, lack sufficient long-term data on their safety, efficacy, and standardized protocols, creating uncertainty around their routine clinical application. Similarly, while shockwave therapy shows promise, its potential in vasculogenic ED remains constrained by variability in protocols and limited long-term evidence, necessitating further high-quality research. The increasing prevalence of ED in younger men highlights the urgent need to explore specific causes, risk factors, and tailored management strategies for this population. Additionally, current guidelines often overlook the integration of psychosexual therapies with pharmacological treatments, despite strong evidence supporting their combined effectiveness in improving patient outcomes. Moreover, disparities in healthcare access and cultural attitudes towards ED remain unexplored, hindering a comprehensive understanding of their impact on diagnosis and treatment. Addressing these gaps will require large-scale, longitudinal studies to refine clinical guidelines and optimize care for diverse patient populations.

Discussion 

Male sexual dysfunctions, particularly ED, are often overlooked and untreated in Asian populations as compared to their European counterparts. This disparity is largely attributed to traditional cultural and religious beliefs, coupled with limited awareness about the condition [113]. Addressing this gap requires eliminating taboos associated with ED, which is a crucial first step in improving its treatment and management.

ED is a multifactorial condition that necessitates a coordinated, multidisciplinary approach to address both its organic and psychological components effectively. A balanced interdisciplinary approach involving urologists, psychiatrists, and other specialists ensures a tailored treatment plan that integrates pharmacological, psychological, and lifestyle interventions, addressing both organic and psychogenic factors of ED. For example, a 45-year-old male presenting with ED and uncontrolled diabetes might require evaluation by a urologist to assess vascular and hormonal factors while a diabetologist optimizes glycemic control, and a psychiatrist addresses underlying depression or anxiety. Similarly, for a 35-year-old patient with performance anxiety-induced ED, a urologist could rule out organic causes and initiate PDE-5I therapy to improve confidence, while a psychologist employs CBT, and relaxation techniques to address anxiety. These scenarios underscore the importance of collaboration among urologists, psychiatrists, endocrinologists, and primary care providers to develop tailored treatment plans that integrate lifestyle modifications, pharmacological therapies, and psychosexual counseling. Regular joint consultations, shared decision-making, and interdisciplinary meetings can significantly enhance treatment outcomes for patients with ED.

Strategies to Eliminate Taboos and Enhance Communication

The stigma surrounding ED, particularly in conservative cultures, such as India, often prevents patients from seeking treatment. This stigma is deeply rooted in societal norms, where ED is perceived as a sign of personal failure or emasculation, further compounding the reluctance to seek medical help. These challenges are more prevalent in rural and semi-urban areas, where patriarchal traditions reinforce these perceptions. Urban areas, however, are beginning to see gradual improvements due to increased awareness and education.

Globally, cultural and societal openness varies significantly. While Western nations like the United States and European nations benefit from comprehensive sex education and societal acceptance, certain ethnic or religious groups within these regions face similar stigmas. Regions like the Middle East and parts of East Asia also share cultural barriers similar to those in India.

Previous research has shown that healthcare providers often find it challenging to discuss sexual well-being with patients [114]. These communication barriers can significantly hinder the timely diagnosis and treatment of ED. To address this, validated questionnaires can be used during consultations to initiate discussions about sexual health and identify issues that might otherwise remain unspoken [115]. These tools provide a structured and less confrontational way for patients to share their concerns, helping bridge the gap between patients and clinicians.

Additionally, targeted awareness campaigns designed in culturally sensitive languages and formats can play a crucial role in normalizing discussions about ED. Such campaigns should consider local traditions and norms, ensuring the message is both relatable and impactful.

Involving local community leaders or influencers to promote open conversations can further enhance their effectiveness. These trusted figures can help dispel myths, educate men about the clinical nature of ED, and emphasize the importance of seeking timely treatment.

By fostering open communication through these strategies, clinicians and public health initiatives can create an environment where ED is treated as a medical condition rather than a source of shame. This approach not only improves patient outcomes but also helps reduce the stigma associated with ED, paving the way for a more inclusive and supportive healthcare landscape.

Diagnosis of Erectile Dysfunction

Diagnosing ED requires a thorough evaluation to identify underlying organic or psychological causes. Risk factors, such as smoking, hypertension, hypogonadism, and medications-induced ED, should be addressed. For most patients, oral pharmacotherapy with PDE-5Is serves as the first-line treatment. Concurrently, addressing comorbidities, such as depression, metabolic syndrome, and cardiovascular disease, is vital to improving overall outcomes.

Treatment: Best Practices

Erectile dysfunction associated with impairment of the nervous system is becoming more prevalent. This rise can be attributed to the complex interplay between ED and other disorders/conditions that affect or disrupt the transmission of signals required for achieving erection. While therapies such as vacuum constriction devices, intracavernosal injections, and transurethral alprostadil have proven effective, their invasive nature can lead to patient dissatisfaction [116]. In contrast, PDE-5Is such as sildenafil, vardenafil, and tadalafil remain the preferred first-line treatment for ED due to their ease of use and effectiveness.

The objectives of ED treatment encompass not only resolving physical symptoms but also restoring patients' quality of life and enabling them to regain satisfying sexual experiences [117]. Comprehensive treatment strategies also include lifestyle modifications, addressing underlying vascular risk factors, and employing a combination of pharmacological and psychosexual therapies to manage both organic and psychosexual dysfunction. For instance, psychological interventions have shown variable success, as demonstrated by an 8-week stress management program that reduced perceived stress and cortisol levels in 31 men with ED though it did not significantly improve erectile function [118].

Referral to a mental health professional is an important consideration for men undergoing treatment for ED. This step can enhance treatment adherence, reduce performance anxiety, and support the integration of therapeutic approaches into the patient’s sexual relationship. Research has consistently demonstrated that combining psychological interventions with PDE-5Is leads to a better outcome. A meta-analysis of 8 studies reported that combination therapy was more effective than PDE-5Is alone (d=0.45, 95% CI = (0.03, 0.89)) [119]. Interventions such as CBT, group therapy, or couples therapy when combined with PDE-5Is, resulted in greater improvements in erectile function compared with PDE-5I alone. However, substantial heterogeneity in these studies underscores the need for further research to identify the most effective psychological interventions [89].

## Conclusions

Erectile dysfunction is a multifactorial condition that requires a multidisciplinary approach, especially when mental health is a contributing factor. Pharmacotherapy, either alone or in combination with graded psychosexual therapy, is highly effective in restoring sexual function for most men. By understanding the psychological factors influencing ED, clinicians can identify high-risk groups and implement early, individualized interventions. Urologists and psychiatrists play pivotal roles in this process, and their proactive engagement through open conversations and reciprocal referrals is essential for comprehensive patient care.
